# Potential of *Aegilops* sp. for Improvement of Grain Processing and Nutritional Quality in Wheat (*Triticum aestivum*)

**DOI:** 10.3389/fpls.2019.00308

**Published:** 2019-03-18

**Authors:** Aman Kumar, Payal Kapoor, Venkatesh Chunduri, Saloni Sharma, Monika Garg

**Affiliations:** National Agri-Food Biotechnology Institute, Mohali, India

**Keywords:** *Aegilops*, grain micronutrients, puroindolins, gliadins, dietary fiber, glutenins, phytochemicals

## Abstract

Wheat is one of the most important staple crops in the world and good source of calories and nutrition. Its flour and dough have unique physical properties and can be processed to make unique products like bread, cakes, biscuits, pasta, noodles etc., which is not possible from other staple crops. Due to domestication, the genetic variability of the genes coding for different economically important traits in wheat is narrow. This genetic variability can be increased by utilizing its wild relatives. Its closest relative, genus *Aegilops* can be an important source of new alleles. *Aegilops* has played a very important role in evolution of tetraploid and hexaploid wheat. It consists of 22 species with C, D, M, N, S, T and U genomes with high allelic diversity relative to wheat. Its utilization for wheat improvement for various abiotic and biotic stresses has been reported by various scientific publications. Here in, for the first time, we review the potential of *Aegilops* for improvement of processing and nutritional traits in wheat. Among processing quality related gluten proteins; high molecular weight glutenins (HMW GS), being easiest to study have been explored in highest number of accessions or lines i.e., 681 belonging to 13 species and selected ones like *Ae. searsii*, *Ae. geniculata* and *Ae. longissima* have been linked with improved bread making quality of wheat. Gliadins and low molecular weight glutenins (LMW GS) have also been extensively explored for wheat improvement and *Ae. umbellulata* specific LMW GS have been linked with wheat bread making quality improvement. *Aegilops* has been explored for seed texture diversity and proteins like puroindolins (*Pin*) and grain softness proteins (*GSP*). For nutrition quality improvement, it has been screened for essential micronutrients like Fe, Zn, phytochemicals like carotenoids and dietary fibers like arabinoxylan and β-glucan. *Ae. kotschyi* and *Ae. biuncialis* transfer in wheat have been associated with higher Fe, Zn content. In this article we have tried to compile information available on exploration of nutritional and processing quality related traits in *Aegilops* section and their utilization for wheat improvement by different approaches.

## Introduction

**Figure 1:**
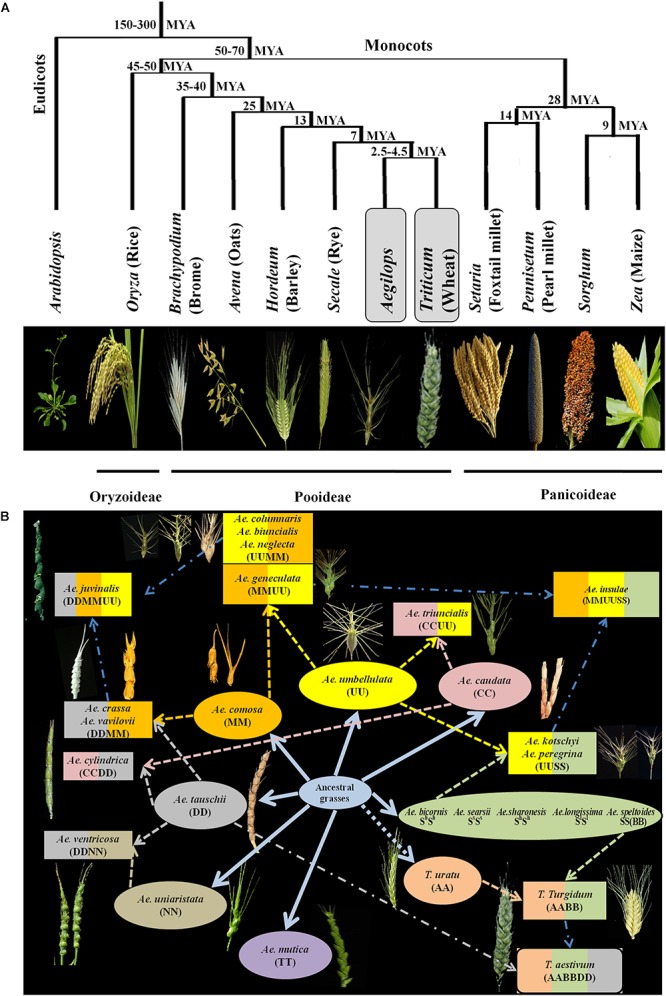
**(A)** Evolutionary relationship among different cereals. *Aegilops* is the closest relative of wheat. Divergence times from a common ancestor are indicated on the branches of the phylogenetic tree in million years (MYA). Modified from Bolot et al. (2009). **(B)** Hypothesized evolution of wheat and species of *Aegilops.* Seven different genomes of *Aegilops* evolved from common ancestor (color coded). Colored dash arrows indicate the involvement of species for formation of other species of *Aegilops*. Blue colored dash dot arrows indicate hypothetical involvement of species. Hypothetical wheat evolution is also explained, cross between *Triticum urartu* and *Ae. speltoides* led to formation of *Triticum turgidum* which further hybridized with *Ae. tauschii* to form cultivated *Triticum aestivum.* Modified from Meimberg et al. (2009).

Some of the most important cereal crops in the world are the members of the grass (Poaceae) family and belong to three major subfamilies – Pooideae, Oryzoideae and Panicoideae. These subfamilies diverged from a common ancestor around 50–70 million years ago (Bolot et al., 2009) (Figure 1A). Genus *Aegilops* is the closest relative of wheat followed by rye, barley, oats and brome in the Pooideae subfamily, rice in Oryzoideae, millets, sorghum and maize in Panicoideae (Figure 1A). Among the Pooideae, wheat (*Triticum* L.) is one of the major staple foods in the world. Due to its unique flour composition and viscoelastic properties, wheat is more suitable for industrialized food production than any other crop. Recently, demand for wheat based convenience foods (fast, ready to eat, frozen etc.) have increased due to the rise in urban population and changing lifestyles. Therefore, the end product quality of wheat has become important. With an increasing concern for texture and taste, there have been a lot of challenges for breeders to develop cultivars that satisfy specific end product requirements. Nutrition is another important aspect of wheat research. There are approximately two billion people in the world that suffer from nutrient deficiency also known as hidden hunger (World Health Organization, 2006). Since wheat provides around one fifth of calorific input to people across the world (Food and Agriculture Organization of United Nations [FAO], 2014), enhancing its nutritive value becomes of great importance.

**Figure 2:**
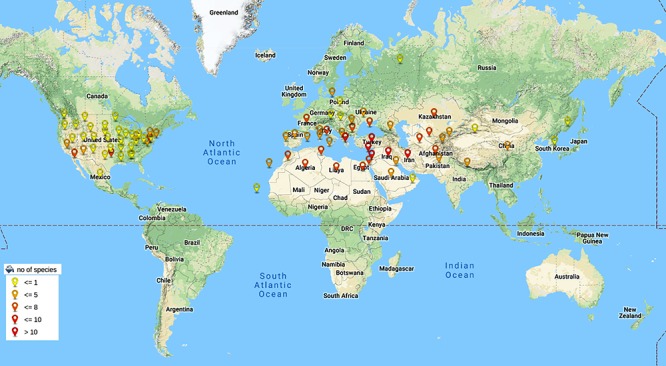
World wide distribution of *Aegilops* species. Species of *Aegilops* are mainly distributed in Eurasia and North America with highest density of occurrence in Fertile Crescent near Middle East (Colors of pins indicate number of species of *Aegilops* found in that area). Data taken from Kew (RBG Grassbase) database (Clayton et al., 2006).

A lot of breeding programs have been initiated to select or develop varieties with improved nutrient content and specific end product quality. The existing germplasm of wheat have been extensively explored for traits related to end product quality and nutrition. The Green revolution has resulted in the development of high yielding and disease resistant varieties and most of the varieties grown today consist of an assembly of genes pyramided by breeders (Lopes et al., 2015). The breeding programs thus have relied on limited number of parent lines for development of wheat germplasm. A report has suggested that due to this genetic bottleneck the population size of wheat has been reduced by 6% (Cavanagh et al., 2013). This narrow genetic diversity often limits the improvement of many traits in wheat. Therefore, the need to explore secondary and tertiary gene pools of wheat has grown. Secondary and tertiary gene pools of wheat mainly consist of wild varieties that are outstanding sources of genetic variability. The secondary gene pool of wheat mainly consists of polyploid *Triticum* and some of *Aegilops* species that share at least one of the A, B and D genomes of hexaploid wheat. The tertiary gene pool consists of wild species with genomes other than A, B and D of wheat. The relationship within and between *Aegilops* and *Triticum* has been a matter of debate and many classification systems exist (Kilian et al., 2011). The latest monograph of Van Slageren (1994) which is based on morphological studies is mostly followed for classification and nomenclature of *Aegilops* and same has been followed in this review article. For wheat the classification system by Dorofeev et al. (1979) is mainly followed. The *Aegilops* genus consists of 11 diploid, 10 tetraploid and 2 hexaploid species (Figure 1B). Species of *Aegilops* occur in Eurasia and North America, but most species are found near the center of origin, the Fertile Crescent in the Middle East, and around the Mediterranean Sea (Figure 2). These species consist of C, D, M, N, S, T and U genomes which have evolved from a common ancestor (Figure 1B) and can be used to incorporate genetic material from the wider gene pool into newly developed cultivars of wheat, thus increasing its genetic diversity.

There have been many reports of species of *Aegilops* being utilized for the improvement of agronomic traits such as rust resistance, powdery mildew resistance and tolerance against other abiotic stresses. More than 41 resistance genes for various biotic and abiotic stresses have been transferred from *Aegilops* to wheat via chromosome translocations or homoeologous recombination (Zhang et al., 2015) and many of these genes have been fairly successful in many breeding programs (Jahier et al., 1989; Ambrozkova et al., 2002; Zhang et al., 2015). This review summarizes the potential of *Aegilops* species for utilization in improvement of end product and nutritional quality of wheat.

## Utilization of *Aegilops* for Improvement of End Product Quality of Wheat

The end product quality of wheat is affected by a number of factors such as: total protein content, grain texture and seed storage proteins composition. Seed storage proteins are the major determinants of end product quality and mainly consist of glutenins and gliadins. A large number of alleles of glutenins and gliadins have been explored in *Aegilops* species with their implications on end product quality. Grain texture related puroindolins, grain softness protein (*GSP*) and many other grain quality related genes have also been reported from *Aegilops*.

### High Molecular Weight Glutenins (HMW GS)

High molecular weight glutenins are the major determinants of bread making quality of wheat. Their importance can be attributed to the fact that though they constitute only about 12% of total seed storage proteins, up to 60% of alterations in baking parameters are affected by them (Payne et al., 1987). HMW GS are coded by *Glu1* loci present on the long arms of homoeologous group 1 chromosomes (1A, 1B and 1D) named as *Glu A1*, *Glu B1* and *Glu D1*, respectively. Each locus produces two subunits of different size; called x-type (larger) and y-type (smaller) subunits i.e., 1A_x_, 1A_y_; 1B_x_, 1B_y_ and 1D_x_, 1D_y_. Subunits 1B_x_, 1D_x_ and 1D_y_ are expressed in most of the bread wheat cultivars while 1B_y_ and 1A_x_ are expressed in some wheat cultivars. The gene coding 1A_y_ generally remains silent in most of bread wheat cultivars (Halford et al., 1989). Only 21 alleles have been reported for *Glu A1* locus, while for *Glu B1* more than 69 alleles and for *Glu D1* only 29 alleles have been documented in bread wheat germplasm (McIntosh et al., 2013).

**Figure 3:**
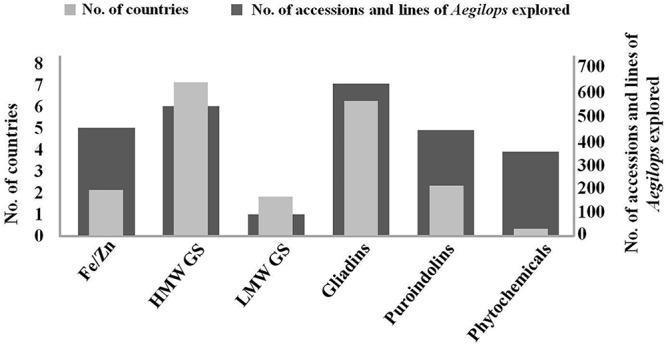
Number of accessions/lines of *Aegilops* explored along with number of countries involved in their exploration for improvement of quality and nutritional traits in wheat. HMW GS are most explored, while phytochemicals are least explored among different groups across the world. Gliadin exploration is being carried out by highest number of countries with LMW and phytochemicals being in the lowest category.

**Figure 4:**
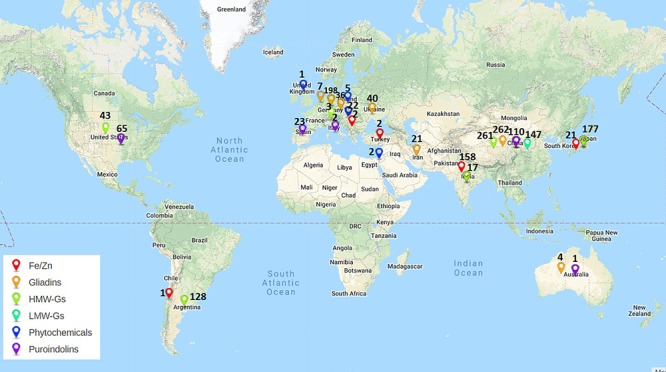
Work done across the world on *Aegilops* for improvement of quality and nutrition. Colors of pins indicate different traits. Numbers along pins indicate total number of accessions/lines explored.

Due to this limited genetic diversity, high levels of allelic variations at *Glu 1* loci are required in the quality wheat breeding practice. These are easiest to study as they can be conveniently resolved and identified by electrophoresis. Among the traits explored here, more than 600 lines and accessions of *Aegilops* have been studied across the world for their rich genetic diversity for HMW GS (Figure 3). Fairly large numbers of countries are involved in the exploration of HMW GS and their distribution across countries is also uniform (Figure 4). Primary structures of most of the *Aegilops* specific HMW GS are similar to wheat subunits. They contain conserved N-, C-terminals and a central variable repetitive region (Mackie et al., 1996; Wan et al., 2000; Xie et al., 2001). More than 30 subunits of HMW GS from *Ae. bicornis*, *Ae. longissima*, *Ae. sharonensis*, *Ae. searsii*, *Ae. cylindrica*, *Ae. umbellulata*, *Ae. caudata*, *Ae. juvenalis*, *Ae. kotschyi, Ae. comosa, Ae. uniaristata, Ae. crassa, Ae. ventricosa* and *Ae. speltoides* have been reported and studied (Table 1) (Wan et al., 2000, 2002; Xie et al., 2001; Liu et al., 2003; Sun et al., 2006; Farkhari et al., 2007; Jiang et al., 2012; Ma et al., 2013). Many of these HMW GS have been cloned and their sequence information is available.

**Table 1:** *Aegilops* species explored for high molecular weight glutenins.

S.No.	Species	Lines/accessions	Subunits	Reference
1.	*Ae. caudata* (CC)	Y588	1C_x_, 1C_y_	Liu et al., 2003
2.	*Ae. caudata* (CC)	Y46	1.1C, 9.1C Increased gluten strength	Du and Zhang, 2017
3.	*Ae. tauschii* (DD)	TD12, TD26, and TD190	DT^1^, DT^2^ Low gluten index, gluten resistance	Hsam et al., 2001
4.	*Ae. tauschii* (DD)	SHW line	2.1^∗^D, 2.1D, 1.5D, 2D, 3D, 4D, 5D, 10D, 10.5D, 12D, 12^∗^D, DT^2^	Pflüger et al., 2001
5.	*Ae. tauschii* (DD)	As2396	13D	Yan et al., 2002
6.	*Ae. tauschii* (DD)	TD159	12.1D	Yan et al., 2004
7.	*Ae. tauschii* (DD)	Multiple accessions	2.1D^,^ 1.5D, 1.5^∗^D, 2D, 3D^,^ 4D, 5.1D, 5D, 5^∗^D 10D, 10.1D, 10.2D, 10.3^∗^D, 10.4D, 11D, 12D, 12.1^∗^D, 12.2^∗^D, DT^2^, 12.3D, 12.4^∗^D, 12.5D	Yan et al., 2003
8.	*Ae. tauschii* (DD)	RM0198, AS2388	2D, 2.1D, 12D	Wan et al., 2005
9.	*Ae. tauschii* (DD)	TD81, TD130	5.1^∗^D, 5^∗^D, 12.1^∗^D, 10.1D	Zhang et al., 2008
10.	*Ae. tauschii* (DD)	TD16	1.6D	An et al., 2009
11.	*Ae. tauschii* (DD)	TD87, TD130, TD151	12.1^∗^D, 12.2D	Zhang et al., 2009
12.	*Ae. tauschii* (DD)	SHW line	2-1D, 2-2D^,^ 2-3D, 1.5-1D,2.1-1D, 10-1D, 12-1D	Xu et al., 2010
13	*Ae. tauschii* (DD)	T67 and T132	3D, 4D	Wang K. et al., 2012
14.	*Ae. bicornis* (S^b^S^b^)	CIae 70	2.9S^b^, 2.3S^b^	Jiang et al., 2012
15.	*Ae. longissima* (S^l^S^l^)	PI 604122	2.9S^l^, 2.3S^l^	Jiang et al., 2012
16.	*Ae. longissima* (S^l^S^l^)	DSL -1S^l^(1B)	2.3^∗^S^l^, 16^∗^S^l^ Improved dough strength and baking quality	Wang S. et al., 2013
17.	*Ae. longissima* (S^l^S^l^)	DSL -1S^l^(1A)	1S^l^_x_, 1S^l^_y_ Higher dough strength, farinograph development time, stability time, gluten index, bread loaf volume, and bread quality score	Garg et al., 2014
18.	*Ae. searsii* (S^s^S^s^)	Multiple accessions	48586S^s^, 48586S^s^, 49077S^s^, 49077S^s^	Sun et al., 2006
19.	*Ae. searsii* (S^s^S^s^)	Multiple DALs	1S^s^_x_, 2S^s^_x_, 1S^s^_y_, 2S^s^_y_ Improved specific sedimentation, mixing properties and polymeric protein content	Garg et al., 2009
20.	*Ae. searsii* (S^s^S^s^)	DSL- GL1402 1B(1S^s^)	2114S^s^, 2114S^s^ Better dough strength and mixing properties	Du et al., 2018
21.	*Ae. sharonensis* (S^sh^S^sh^)	PI 584388	2.9S^sh^, 2.3S^sh^	Jiang et al., 2012
22.	*Ae. speltoides* (SS)	Multiple accessions	15^∗^S_x,_ 15^∗^S_y_	Ma et al., 2013
23.	*Ae. umbellulata* (UU)	IG46953, Y39, Y137, and Y139	1U_x_, 1U_y_	Liu et al., 2003
24.	*Ae. cylindrica* (CCDD)	Multiple accessions	1C_x_, 1C_y_	Wan et al., 2000
25.	*Ae. biuncialis* (U^b^U^b^M^b^M^b^)	DAL1U^b^	1U_x_, 1U_y_ Increased protein content, Zeleny sedimentation value, wet gluten content, and grain hardness	Zhou et al., 2014
26.	*Ae. geniculata* (MMUU)	Multiple DALs DSLs- 1M^g^(1A), 1M^g^(1B), 1M^g^(1D)	1U^g^_x,_ 1U^g^_y_ 1M^g^_x_, 1M^g^_y_	Garg et al., 2016
27.	*Ae. kotschyi* (UUSS)	Multiple accessions	2.3U/S_x_, 1^∗^U/S_x_, 3^∗^U/S_x_, 20^∗^U/S_y_, 8^∗^U/S_y_	Ma et al., 2013
28.	*Ae. kotschyi* (UUSS)	Wheat*- Ae. kotschyi* acc. 396 derivative 49-1-73-10	1U_x_, 1U_y_	Singh et al., 2016
29.	*Ae. juvenalis* (DDMMUU)	Not mentioned	1J_x_,2J_x_,1J_y_,2J_y_	Xie et al., 2001

*SHW – Synthetic hexaploid wheat, DALs – Disomic addition lines, DSLs – Disomic substitution lines*.

*Aegilops tauschii* is regarded as D genome donor of wheat and its many accessions for HMW GS have been explored. For HMW GS, extensive studies have been done on the *Glu D1* loci from *Ae. tauschii* as variation in this locus is very important in determining dough strength and other end product qualities. More than 40 HMW GS allelic variants have been reported from multiple accessions of *Ae. tauschii* (Yan et al., 2002, 2003, 2004; Wan et al., 2005; Zhang et al., 2008; An et al., 2009; Wang K. et al., 2012). Many D genome synthetic hexaploids have been generated by crossing tetraploid durum wheat with *Ae. tauschii* and thus HMW GS alleles 2.1^∗^D, 2.1D, 1.5D, 2D, 3D, 4D, 5D, 10D, 10.5D, 12D, 12^∗^D, T2 (Pflüger et al., 2001), 2-1D, 2-2D, 2-3D, 1.5-1D, 2.1-1D, 10-1D, and 12-1D (Xu et al., 2010) have been transferred to wheat. D genome specific subunits of 5D_x_+10D_y_ have been reported to be most important for bread making quality of wheat (Branlard and Dardevet, 1985b). Attempts have been made to replace null *Glu A1* allele of wheat with *Glu D1* allele carrying 5D_x_+10D_y_ subunits (Ceoloni et al., 1996; Ammar et al., 1997). Substitution of chromosome 1A with 1D has shown improvement in dough strength (Liu and Shepherd, 1995; Garg et al., 2007). A chromosomal translocation line 1AS.1AL-1DL carrying *Glu D1d* alleles (5D_x_+10D_y_) was generated in durum wheat background and was reported to possess improved mixing properties (Klindworth et al., 2005). Transfer of *Glu D1* locus to chromosome 1R and 1A of *Triticale* has also been shown to improve bread making properties (Lukaszewski, 2006).

Implications of many HMW GS from *Aegilops* species on product quality have been studied. Subunits 1.1C and 9C from *Ae. caudata* led to increased gluten strength (Du and Zhang, 2017) while 2D+T1+T2 subunits from *Ae. tauschii* are associated with low gluten index and gluten resistance (Hsam et al., 2001). Disomic addition lines (DALs) from *Ae. searsii* have been used to transfer HMW GS subunits 1S^s^_x_1, 1S^s^_x_2, 1S^s^_y_1and 1S^s^_y_2 into wheat (Garg et al., 2009). These addition lines showed improved specific sedimentation, mixing properties and polymeric protein content. Similarly, DAL-1U^b^ of *Ae. biuncialis* (Zhou et al., 2014) were generated to transfer 1U^b^_x_ and 1U^b^_y_ subunits to wheat and these lines showed increased protein content, Zeleny sedimentation value, wet gluten content, and grain hardness. Addition lines of *Ae. umbellulata* showed negative impact of its HMW GS on dough strength (Garg et al., 2009). Addition of 1U^g^ chromosome to transfer 1U^g^_x_ and 1U^g^_y_ subunits from *Ae. geniculata* led to reduced dough strength (Garg et al., 2016). Addition of 1M^g^ chromosome from *Ae. geniculata* to Chinese Spring background of wheat improved dough strength significantly (Garg et al., 2016). Many disomic substitution lines (DSLs) have also been generated from DALs. Addition line of 1M^g^ chromosome from *Ae. geniculata* was used to generate chromosome specific DSLs- 1M^g^(1A), 1M^g^(1B) and 1M^g^(1D). DSLs- 1M^g^(1A) and 1M^g^(1B) showed improved dough strength and mixing properties but 1M^g^(1D) showed reduced dough strength (Garg et al., 2016). Substitution of chromosome 1S^l^ from *Ae. longissima* with chromosomes 1A (Garg et al., 2014) and 1B (Wang S. et al., 2013) significantly improved bread making qualities of wheat. Similarly substituting chromosome 1S^s^ from *Ae. searsii* with 1B led to better dough strength and mixing properties (Du et al., 2018). All these addition and substitution lines that improved dough strength can be utilized to transfer HMW GS alleles into wheat in form of fine translocations with least linkage drag.

### Low Molecular Weight Glutenins (LMW GS)

Low molecular weight glutenins account for 60% of total glutenins and one third of seed storage proteins. Genes that code for LMW GS (*Glu A3*, *Glu B3* and *Glu D3)* are present on the short arms of group 1 homoeologous chromosomes (Singh and Shepherd, 1988; Sreeramulu and Singh, 1997). Only six alleles at *Glu A3*, nine at *Glu B3* and five at *Glu D3* have been reported in wheat germplasm (McIntosh et al., 2013). There are additional three loci (*Glu 2*, *Glu 4* and *Glu 5)* present on chromosomes *1B*, *1D* and *7D* (Jackson et al., 1985; Liu and Shepherd, 1995; Sreeramulu and Singh, 1997). On the basis of SDS PAGE mobility LMW GS can be classified into B, C and D types (Jackson et al., 1983). B type LMW GS are further classified into m, s and i type on the basis of first amino acid methionine, serine and isoleucine, respectively (Muccilli et al., 2010). Besides these three types, a novel LMW GS, l type was identified specifically in *Aegilops* with first amino acid being leucine (Wang K. et al., 2011).

Low molecular weight glutenins provide viscoelastic properties to the dough and some of their alleles have been reported to be associated with good bread making quality. *Aegilops* species serve as rich source of genetic diversity of LMW GS. More than 13 alleles of LMW GS from *Ae. tauschii* (Pei et al., 2007; Zhao et al., 2008; Cao et al., 2018), 12 alleles from *Ae. longissima* (Jiang et al., 2008; Huang et al., 2010a), 11 alleles from *Ae. comosa* (Wang K. et al., 2011), 4 alleles from *Ae. neglecta* (Li X. et al., 2008), 3 alleles from *Ae. umbellulata* and one from *Ae. kotschyi* (Li X. et al., 2008), *Ae. uniaristata*, *Ae. caudata* and *Ae. speltoides* each (Table 2) (Li et al., 2010) have been identified and characterized (Table 2). Most of these LMW GS genes have been cloned and their sequence information is available in NCBI. There is large amount of variability present in *Aegilops* specific LMW GS. *Ae. tauschii* exhibits even greater variation in LMW GS sequences than wheat (Rehman et al., 2008). There have been reports of novel LMW GS genes *Glu U3a* and *Glu U3b* from wheat-*Ae. umbellulata* 1U(1B) substitution line showing improved bread making and mixing properties. This substitution line was used to transfer the LMW GS genes to wheat. The line thus developed showed improvement in dough development time, stability time, farinograph quality number, gluten index, loaf size and inner structure (Wang et al., 2017). The variability in LMW GS genes found in *Aegilops* species indicates a large potential for their utilization in improvement of end product qualities of wheat. In comparison to HMW GS, works on transfer of LMW GS alleles from *Aegilops* species to wheat cultivars have been limited. As per literature only 147 accessions/lines have been explored for LMW GS, which too mainly in China (Figure 3, 4) and further exploration is needed.

**Table 2:** *Aegilops* species explored for low molecular weight glutenins.

S.No.	Species	Lines/accessions	Characteristics	Reference
1	*Ae. caudata* (CC)	PI254863	*AmLMW-m1*	Li et al., 2010
2	*Ae. tauschii* (DD)	T121, T128, T132	*LMW-T121*, *LMW-T128*, *LMW-T132*	Pei et al., 2007
3	*Ae. tauschii* (DD)	Multiple accessions	*GluD^t^3-3*, *GluD^t^3-6*	Zhao et al., 2008
4	*Ae. tauschii* (DD)	Multiple accessions	*TaALPb7D-(A–M)*	Cao et al., 2018
5	*Ae. comosa* (MM)	PI551017	*AcLMW-m1*	Li et al., 2010
6	*Ae. comosa* (MM)	PI 551017, PI 551019	*AcLMW-L1*, *AcLMW-L2*, *AcLMW-L3*, *AcLMW-L4*, *AcLMW-I1*, *AcLMW-I2*, *AcLMW-I3*, *AcLMW-M1*, *AcLMW-M2*, *AcLMW-M3*	Wang K. et al., 2011
7	*Ae. uniaristata* (NN)	PI554419	*AuLMW-m1*	Li et al., 2010
8	*Ae. speltoides* (SS)	PI170204	*AsLMW-m1*	Li et al., 2010
	*Ae. longissima* (S^l^S^l^)	PI604108, PI604110	*TzLMW-m1*, *TzLMW-m2*, *TdLMW-m1 AlLMW-m2*	Jiang et al., 2008
9	*Ae. longissima* (S^l^S^l^)	PI604103, PI604124, PI604126, PI604129	*SL124-1*, *SL126-1*, *SL129-1*, *SL129-2*, *SL129-3*, *SL129-4*, *SL103-1*, *SL103-2*	Huang et al., 2010a
11	*Ae. umbellulata* (UU)	PI222762	*AumLMW-m1*	Li et al., 2010
12	*Ae. umbellulata* (UU)	DSL -1U(1B)	*Glu-U3a*, *Glu-U3b* Improved dough development time, stability time, farinograph quality number, gluten index, loaf size and inner structure	Wang et al., 2017
13	*Ae. umbellulata* (UU)	CNU609 [CS- DSL 1U(1B) derivative]	*Glu-U3a*, *Glu-U3b* Improved dough development time, stability time, farinograph quality number, gluten index, loaf size and inner structure	Wang et al., 2017
14	*Ae. neglecta* (UUMM)	PI298897	*AnLMW-m1*, *AnLMW-m2*, *AnLMW-m3*, *AnLMW-m4*	Li X. et al., 2008
15	*Ae.kotschyi* (UUSS) *Ae. juvenalis* (DDMMUU)	PI226615, PI330485	*AjkLMW-I*	Li X. et al., 2008

*DSL – Disomic substitution line, ALP – Avenin like protein*.

### Gliadins

Gliadins account for 40–50% of total seed storage proteins. They have impacts on both processing and nutritional quality. Gliadins can be separated into α-/β-, γ-, and ω-gliadins based on differences in their mobility on SDS PAGE gel. *Gli 1* loci present on short arms of homoeologous group 1 chromosomes code for all ω- and most of γ-gliadins, while, *Gli 2* loci on the short arms of homoeologous group 6 chromosomes code for all α-, most of the β-, and some of the γ-gliadins (Payne, 1987; Metakovsky et al., 1990; Metakovsky, 1991). The effect of gliadins on rheological properties of dough has been studied (Branlard and Dardevet, 1985a). Due to lack of free cysteine residues in most of the gliadins, they are unable to form intermolecular S-S linkages. Hence, their overall impact on processing quality is small as compared to glutenins (Qi et al., 2009). Gliadins may act as chain terminators for gluten polymer. They therefore might limit the size of gluten complex and hence affect end product quality (Muccilli et al., 2005). However, many gliadins with odd number of cysteins also exist (Anderson et al., 2001; Goryunova et al., 2012). So some gliadins might also participate in gluten polymerization. It has been hypothesized that gliadins proteins contribute mostly toward dough cohesiveness (Uthayakumaran et al., 2000) and viscosity (Pistón et al., 2011) rather than resistance and extension. Studies on effect of *Aegilops* specific gliadins on product quality are limited. Multiple accessions of *Ae. biuncialis* and *Ae. umbellulata* have been reported to possess high gluten quality indices due to gliadins (Ahmadpoor et al., 2014) (Table 3). Gliadins from *Ae. cylindrica* (Khabiri et al., 2013), *Ae. biuncialis* (Kozub et al., 2012) and *Ae. geniculata* (Medouri et al., 2015) have been characterized on the basis of mobility on SDS PAGE (Table 3). Many ω-gliadins have been sequenced and characterized from *Ae. tauschii* (Yan et al., 2003; Hassani et al., 2009). γ-gliadins have been characterized from *Ae. caudata, Ae. uniaristata, Ae. mutica, Ae. umbellulata* (Goryunova et al., 2012), *Ae. bicornis, Ae. searsii, Ae. sharonensis* (Qi et al., 2009; Huang et al., 2010b), *Ae. longissima* (Qi et al., 2009), *Ae. tauschii* (Qi et al., 2009; Goryunova et al., 2012; Wang S. et al., 2012), *Ae. speltoides* (Huang et al., 2010b; Goryunova et al., 2012), *Ae. markgrafii* (Li M. et al., 2017) and *Ae. cylindrica* (Wang S. et al., 2012) (Table 3).

**Table 3:** *Aegilops* species explored for gliadins.

S. No.	Species	Lines/accessions	Characteristics	Reference
1	*Ae. caudata* (CC)	κ-2255	γ-gliadins	Goryunova et al., 2012
2	*Ae. caudata* (CC)	PI573416, PI551119, PI298889, PI564196	α-gliadins	Li et al., 2012
3	*Ae. caudata* (CC)	Y46	γ-gliadins	Li M. et al., 2017
4	*Ae. tauschii* (DD)	Multiple accessions	ω-Gliadins	Yan et al., 2003
5	*Ae. tauschii* (DD)	AUS18913, CPI110856	ω-gliadin γ-gliadin	Hassani et al., 2009
6	*Ae. tauschii* (DD)	AS60	γ-gliadins	Qi et al., 2009
7	*Ae. tauschii* (DD)	AUS18913, CPI110856	ω-gliadin	Hassani et al., 2009
8	*Ae. tauschii* (DD)	T15, T43, T26	α-gliadins	Xie et al., 2010
9	*Ae. tauschii* (DD)	κ-1368	γ-gliadins	Goryunova et al., 2012
10	*Ae. tauschii* (DD)	AT9, AT9.1, AT25, AT48, AT176	γ-gliadins	Wang S. et al., 2012
11	*Ae. tauschii* (DD)	T006	α-gliadins	Li Y.G. et al., 2017
12	*Ae. comosa* (MM)	PI551020	α-gliadins	Li et al., 2012
13	*Ae. uniaristata* (NN)	κ-650	γ-gliadins	Goryunova et al., 2012
14	*Ae. uniaristata* (NN)	PI276996, PI276996, PI554420, PI554418	α-gliadins	Li et al., 2012
15	*Ae. bicornis* (S^b^S^b^)	CIae 47	γ-gliadins	Qi et al., 2009
16	*Ae. bicornis* (S^b^S^b^)	CIae 47, CIae 70	γ-gliadins	Huang et al., 2010c
17	*Ae. bicornis* (S^b^S^b^)	CIae 47	α-gliadins	Huang et al., 2016
18	*Ae. longissima* (S^l^S^l^)	PI 604104	γ-gliadins	Qi et al., 2009
19	*Ae. longissima* (S^l^S^l^)	PI 604104, PI604129, PI604130, PI604131, PI604133	γ-gliadins	Huang et al., 2010c
20	*Ae. searsii* (S^s^S^s^)	PI 599123	γ-gliadins	Qi et al., 2009
21	*Ae. searsii* (S^s^S^s^)	PI 599122, PI599124, PI599138, PI599139, PI599150	γ-gliadins	Huang et al., 2010c
22	*Ae. searsii* (S^s^S^s^)	Multiple accessions	α-gliadins	Huang et al., 2016
23	*Ae. sharonesis* (S^sh^S^sh^)	CIae 32	γ-gliadins	Qi et al., 2009
24	*Ae. sharonensis* (S^sh^S^sh^)	PI584350	α-gliadins	Huang et al., 2010b
25	*Ae. sharonensis* (S^sh^S^sh^)	CIae 32, PI 584345, PI 584349, PI584350, PI584357, PI584391	γ-gliadins	Huang et al., 2010c
26	*Ae. sharonensis* (S^sh^S^sh^)	Multiple accessions	α-gliadins	Huang et al., 2016
27	*Ae. speltoides* (SS)	PI 584391, PI554305, PI560527	γ-gliadins	Huang et al., 2010c
28	*Ae. speltoides* (SS)	CGN10682, CGN10684	γ-gliadins	Goryunova et al., 2012
29	*Ae. umbellulata* (UU)	κ-1588	γ-gliadins	Goryunova et al., 2012
30	*Ae. umbellulata* (UU)	PI298906, PI542364, PI573516	α-gliadins	Li et al., 2012
31	*Ae. mutica* (TT)	κ-1581	γ-gliadins	Goryunova et al., 2012
32	*Ae. cylindrica* (CCDD)	PI256029	γ-gliadins	Wang S. et al., 2012
32	*Ae. cylindrica* (CCDD)	Multiple accessions	Gliadins	Khabiri et al., 2013
34	*Ae. geniculata* (MMUU)	Multiple accessions	Gliadins	Medouri et al., 2015
35	*Ae. biuncialis* (U^b^U^b^M^b^M^b^)	Multiple accessions	Gliadins	Kozub et al., 2012

Although fairly large number of lines and accessions (more than 400) of *Aegilops* have been explored for gliadins (Figure 3) and their exploration is quite distributed across several countries of the world (Figure 4), most of the research conducted on gliadins of *Aegilops* is related to identification and characterization of allergic epitopes of celiac disease (Juhász et al., 2018). α-Gliadins are considered to be most allergic and are mostly responsible for inflammatory responses to celiac disease. α-Gliadins from *Ae. speltoides* (Spaenij-Dekking et al., 2004) and *Ae. tauschii* (Xie et al., 2010; Li et al., 2012, 2013) have been reported to be less allergic than corresponding wheat alleles. Novel α-gliadins have been reported from *Ae. bicornis, Ae. searsii, Ae. sharonensis* (Huang et al., 2010c, 2016), *Ae tauschii* (Xie et al., 2010; Li Y.G. et al., 2017), *Ae. comosa, Ae. umbellulata, Ae. markgrafii* and *Ae. uniaristata* (Li et al., 2012) (Table 3). These gliadins could contain useful variation and can be replaced from more allergic gliadins in wheat.

### Puroindolins and Grain Softness Protein

Grain texture plays important role in determining end product quality of wheat. Soft textured wheat is mostly used for pastries and biscuits, while hard textured wheat is used in making bread, pasta and noodles (Morris and Rose, 1996). Grain texture is determined by the hardness (*Ha*) locus present on the telomeric region of short arm of chromosome 5D of wheat which contains ten tightly linked genes (Chantret et al., 2005). Among them, three genes- *puroindolin a* (*Pin a*), *puroindolin b* (*Pin b*) and *grain softness protein-1* (*GSP*) play major role in determining seed texture. These three genes code for the proteins which constitute a 15 kDa complex- friabilin, with *Pin a*, *Pin b* as major components and *GSP-1* as minor component (Cuesta et al., 2015). This protein complex is found abundantly on the surface of starch granules of soft textured wheat and in very small amounts in hard textured wheat (Chen et al., 2005). Presence of this complex results in prevention of adhesion between starch granules and gluten matrix and hence soft texture (Greenwell and Schofield, 1986). *Pin a* and *Pin b* genes have also been associated with antimicrobial properties conferring protection to seed (Dubreil et al., 1998; Miao et al., 2012). *Pin a* especially has been hypothesized to have evolved in response to plant pathogens to enhance plant fitness (Massa and Morris, 2006). Soft seed texture is associated with wild type alleles of *Pin a* and *Pin b* (*Pina*-*D1a* and *Pinb*-*D1a*) and many mutations in those alleles have been linked with hard texture (Giroux and Morris, 1998). *Pin a* and *Pin b* genes are not present on A and B genome specific chromosomes (Li W. et al., 2008) and diploid species with A and B genomes as well as tetraploid durum wheat lack them, as a result of which durum has a very hard kernel texture (Chen et al., 2005). This also indicates *Ae. tauschii* as the donor of *Pin* genes in hexaploid wheat. Species of *Aegilops* have been explored for presence of different *Pin* alleles. Many novel *Pin* alleles have been reported from multiple accessions of *Ae. tauschii* (Table 4) (Massa et al., 2004; Gazza et al., 2006; Simeone et al., 2006; Liu et al., 2016). Many accessions of *Ae. tauschii* have been crossed with tetraploid durum wheat to produce synthetic wheat lines with different textures (Reynolds et al., 2010; Li et al., 2007). Many other *Aegilops* species have also been explored for variability in *Pin a* and *Pin b* gene alleles.19 alleles of puroindolins from *Ae. speltoides*, 9 alleles from *Ae. searsii*, 8 alleles from *Ae. comosa*, 7 from *Ae. caudata* and *Ae. umbellulata* each, 4 from *Ae. longissima, Ae. ventricosa* and *Ae. bicornis* each and 2 from *Ae. sharonensis* have been reported (Table 4) (Gazza et al., 2006; Simeone et al., 2006; Cuesta et al., 2013, 2015).

**Table 4:** *Aegilops* species explored for puroindolins and grain softness proteins.

S.No.	Species	Source	*Pin a* Alleles	*Pinb* Alleles	*GSP* Alleles	Reference
1	*Ae. caudata* (CC)	Multiple accessions	*Pina-C1-I*,	*Pinb-C1-I*,		Cuesta et al., 2013
			*Pina-C1-II*,	*Pinb-C1-II*,		
			*Pina-C1-III*,	*Pinb-C1-III*		
				*Pinb-C1-IV*		
2	*Ae. caudata* (CC)	Multiple accessions			*GSP-C1-I*,	Cuesta et al., 2015
					*GSP-C1-II*,	
					*GSP-C1-III*,	
					*GSP-C1-IV*	
3	*Ae. tauschii* (DD)	CPI110799	*Pina*	*Pinb*	*GSP*	Turnbull et al., 2003
4	*Ae. tauschii* (DD)	Multiple accessions	*Pina-D1g*,	*Pinb-D1i*,	*GSP-D1g*,	Massa et al., 2004
			*Pina-D1a*,	*Pinb-D1j*,	*GSP-D1h*,	
			*Pina-D1c*,	*Pinb-D1h*,	*GSP-D1c*,	
			*Pina-D1d*,	*Pinb-D1a*	*GSP-D1e*	
			*Pina-D1e*,		*GSP-D1d*	
			*Pina-D1f*,		*GSP-D1f*,	
					*GSP-D1b*	
5	*Ae. tauschii* (DD)	TA1704, TA1691, TA2381, TA10	*Pina-D1d*,	*Pinb-D1i*,		Simeone et al., 2006
			*Pina-D1a*,	*Pinb-D1j*,		
			*Pina-D1c*	*Pinb-D1h*		
6	*Ae. tauschii* (DD)	L35	*Pina-D1d*	*Pinb-D1i*		Gazza et al., 2006
7	*Ae. tauschii* (DD)	SHW	*Pina-D1a*,	*Pinb-D1h*,		Li et al., 2007
			*Pina-D1c*	*Pinb-D1j*		
8	*Ae. tauschii* (DD)	SHW	*Pina-D1c*	*Pinb-D1h*		Reynolds et al., 2010
9	*Ae. tauschii* (DD)	Multiple accessions	*Pina-D1o*	*Pinb-D1dt*,		Liu et al., 2016
				*Pinb-D1it*		
10	*Ae. comosa* (MM)	Multiple accessions	*Pina-M1-I*,	*Pinb-M1-I*,		Cuesta et al., 2013
			*Pina-M1-II*,	*Pinb-M1-II*		
			*Pina-M1-III*	*Pinb-M1-III*,		
				*Pinb-M1-IV*,		
				*Pinb-M1-V*		
11	*Ae. comosa* (MM)	Multiple accessions			*GSP-M1-I*,	Cuesta et al., 2015
				*GSP-M1-II*		
12	*Ae. speltoides* (SS)	TA2368, TA1789, TA1777	*Pina-S1c*,	*Pinb-S1c*,		Simeone et al., 2006
			*Pina-S1d*,	*Pinb-S1d*,		
			*Pina-S1e*	*Pinb-S1e*		
13	*Ae. speltoides* (SS)	Multiple accessions	*Pina-S^1^-I*,	*Pinb-S^1^-I*,		Cuesta et al., 2013
			*Pina-S^1^-II*,	*Pinb-S^1^-II*,		
			*Pina-S^1^-III*,	*Pinb-S^1^-III*,		
			*Pina-S^1^-IV*	*Pinb-S^1^-IV*,		
				*Pinb-S^1^-V*,		
				*Pinb-S^1^-VI*,		
				*Pinb-S^1^-VII*,		
				*Pinb-S^1^-VIII*,		
				*Pinb-S^1^-IX*		
14	*Ae. speltoides* (SS)	Multiple accessions			*GSP-S1-I*,	Cuesta et al., 2015
					*GSP-S1-II*,	
					*GSP-S1-III*,	
					*GSP-S1-IV*,	
					*GSP-S1-V*,	
					*GSP-S1-VI*	
					*GSP-S1-VII*	
15	*Ae. searsii* (S^s^S^s^)	TA1837, TA2355	*Pina-S^s^1a*,	*Pinb-S^s^1b*,		Simeone et al., 2006
			*Pina-S^s^1b*	*Pinb-S^s^1a*		
16	*Ae. searsii* (S^s^S^s^)	Multiple accessions	*Pina-S^s^1-I*,	*Pinb-S^s^1-I*,		Cuesta et al., 2013
			*Pina-S^s^1-II*	*Pinb-S^s^1-II*,		
				*Pinb-S^s^1-III*		
17	*Ae. searsii* (S^s^S^s^)	Multiple accessions			*GSP-S^s^1-I*,	Cuesta et al., 2015
				*GSP-S^s^1-II*		
18	*Ae. longissima* (S^l^S^l^)	TA1912, TA1921,	*Pina-S^l^1a*,	*Pinb-S^l^1a*,		Simeone et al., 2006
			*Pina-S^l^1b*	*Pinb-S^l^1b*		
19	*Ae. bicornis* (S^b^S^b^)	TA1954, TA1942	*Pina-S^b^1a*,	*Pinb-S^b^1a*,		Simeone et al., 2006
			*Pina-S^b^1b*	*Pinb-S^b^1b*		
20	*Ae. sharonensis* (S^h^S^h^)	TA1999	*Pina-S^sh^1a*	*Pinb-S^sh^1a*		Simeone et al., 2006
21	*Ae. umbellulata* (UU)	Multiple accessions	*Pina-U1-I*,	*Pinb-U1-I*,		Cuesta et al., 2013
			*Pina-U1-II*,	*Pinb-U1-II*,		
			*Pina-U1-III*,	*Pinb-U1-III*		
				*Pina-U1-IV*		
22	*Ae. umbellulata* (UU)	Multiple accessions			*GSP-U1-I*,	Cuesta et al., 2015
				*GSP-U1-II*,		
				*GSP-U1-III*,		
				*GSP-U1-IV*		
23	*Ae. ventricosa* (DDNN)	L36	*Pina-D1a*,	*Pinb-D1h, and*		Gazza et al., 2006
			*Pina-N1a*	*Pinb-N1a.*		

*SHW – Synthetic hexaploid wheat*.

Unlike *Pin a* and *Pin b, GSP* genes are present on A and B genome specific chromosomes (5A, 5B). However, their deletion does not impact the grain texture (Chen et al., 2005). *GSP* genes have been characterized in many species of *Aegilops.* Many novel *GSP* alleles in *Ae. tauschii*, *Ae. comosa, Ae. caudata, Ae. searsii, Ae. speltoides* and *Ae. umbellulata* have been reported and characterized (Massa et al., 2004; Cuesta et al., 2015). Almost 100 alleles of *Pin a*, *Pin b* and *GSP* have been identified across 200 lines/accessions of *Aegilops* (Figure 3). Their exploration is quite uniform across different countries in the world (Figure 4). All these alleles can serve as useful source of variation and need to be evaluated and utilized in breeding programs for extending the textural characteristics of wheat.

## Utilization of *Aegilops* for Improvement of Nutritional Quality of Wheat

Improvement of nutrition is a very important aspect of wheat research as there are over two billion people worldwide, suffering from deficiencies in proteins and micronutrients (World Health Organization, 2006). Nutritive value of wheat can be enhanced by increasing micronutrients like Fe and Zn, protein content, dietary fibers and many other phytochemicals such as carotenoids, vitamins etc. *Aegilops* genus can serve as important source for enhancing nutrition in wheat due to its high genetic variability.

### Improvement of Grain Micronutrients Concentration

Micronutrients play very important role as health promoting factors. Since most of the world’s population especially developing nations depend on cereal based diet to fulfill their micronutrients requirements, it becomes very important to develop the varieties with improved micronutrients content. Iron and zinc are the most important components among micronutrients. Most varieties of wheat lack sufficient levels of iron and zinc due to low genetic variability. To overcome this limited genetic variability more than 180 lines/accessions of *Aegilops* have been explored (Figure 2, 3). Many accessions of *Ae. kotschyi* (Chhuneja et al., 2006; Rawat et al., 2009a,b, 2011), *Ae. longissima* (Kumari et al., 2013), *Ae. tauschii*, *Ae. peregrina*, *Ae. cylindrica*, *Ae. ventricosa* and *Ae. geniculata* (Rawat et al., 2009b) have been reported to have higher contents of iron and zinc in seeds (Table 5). These accessions can be exploited for increasing grain iron and zinc content. Amphiploids (Tiwari et al., 2010) and partial amphiploids (Rawat et al., 2009b) generated by crossing *Ae. kotschyi* accessions with wheat have been reported to have higher grain iron and zinc content. Many disomic and monosomic addition lines specific to various *Aegilops* species have been explored for higher micronutrient content. Fair exploration of grain micronutrient content has been carried out in many countries (Figure 3). Major exploration of *Aegilops* for Fe/Zn is from India (158 lines and accessions) as compared to other countries (Figure 4). Many disomic and monosomic addition lines of *Ae. peregrina*, *Ae. longissima* and *Ae. umbellulata*, in wheat have been explored for grain iron and zinc concentrations (Kumari et al., 2012). Addition of chromosome pairs 1S^l^ (Wang S. et al., 2011), 2S^l^ (Wang S. et al., 2011; Kumari et al., 2012) and 7S^l^ (Wang S. et al., 2011) of *Ae. longissima* into wheat showed increase in grain iron and zinc content. Similarly, DALs of chromosomes 2S^v^, 2U^v^, 7U^v^ (Kumari et al., 2012) and 4S^v^ (Wang S. et al., 2011) of *Ae. peregrina*, 2U (Kumari et al., 2012) and 6U (Wang S. et al., 2011; Kumari et al., 2012) of *Ae. umbellulata*, 1S^s^ and 2S^s^ of *Ae. searsii* (Wang S. et al., 2011), 5M^g^ of *Ae. geniculata* (Wang S. et al., 2011) and B chromosome additions from *Ae. caudata* (Wang S. et al., 2011) have been reported to increase the iron and zinc content in grains (Table 5). The addition lines can be used to produce DSLs which are better materials to study the compensation effect of alien chromosomes into wheat. Substitution of 4B chromosome of wheat with 3M^b^ chromosome of *Ae. biuncialis* (Farkas et al., 2014) also lead to increased iron and zinc content. Similarly, 2S(2A), 7U(7A) substitutions specific to *Ae. kotschyi* (Tiwari et al., 2010) have been reported with increased grain iron and zinc content.

**Table 5:** *Aegilops* species explored for grain micronutrient content.

S.No.	*Aegilops* sp.	Lines/Accessions	Trait	Reference
1.	*Ae. caudata* (CC)	DALs	Iron, Zinc	Wang S. et al., 2011
2.	*Ae. tauschii* (DD)	SHW	Zn uptake	Cakmak et al., 1999
3.	*Ae. tauschii* (DD)	SHW	Iron, Manganese, Zinc, Calcium, Uptake of Iron, Manganese, Potassium, Phosphorus	Calderini and Ortiz-Monasterio, 2003
4.	*Ae. tauschii* (DD)	SHW	Iron, Zinc	Chhuneja et al., 2006
5.	*Ae. longissima* (S^l^S^l^)	DALs 1S^l^, 2S^l^	Iron, Zinc	Wang S. et al., 2011
6.	*Ae. longissima* (S^l^S^l^)	2S^l^, 7S^l^	Iron, Zinc	Kumari et al., 2012
7.	*Ae. longissima* (S^l^S^l^)	DALs	Iron, Zinc, Copper, Manganese, Calcium, Magnesium, Potassium	Kumari et al., 2012
8.	*Ae. longissima* (S^l^S^l^)	Wheat – *Ae. longissima* derivatives	Iron, Zinc	Sharma et al., 2014
9.	*Ae. longissima* (S^l^S^l^)	Hybrids	Iron, Zinc	Tiwari et al., 2008
10.	*Ae. searsii* (S^s^S^s^)	DALs 1S^s^, 2S^s^	Iron, Zinc	Wang S. et al., 2011
11.	*Ae. umbellulata* (UU)	DALs 2U, 6U	Iron, Zinc	Wang S. et al., 2011
12.	*Ae. umbellulata* (UU)	DAL 2U	Iron, Zinc	Kumari et al., 2012
13.	*Ae. cylindrica* (CCDD)	DALs	Iron, Zinc	Rawat et al., 2009a
14.	*Ae. cylindrica* (CCDD)	Accessions and interspecific hybrids with *Triticum aestivum*	Iron, Zinc	Rawat et al., 2009a
15.	*Ae. ventricosa* (DDNN)	DALs	Iron, Zinc	Rawat et al., 2009b
16.	*Ae. ventricosa* (DDNN)	Accessions and interspecific hybrids with *Triticum aestivum*	Iron, Zinc	Rawat et al., 2009a
17.	*Ae. geniculata* (MMUU)	Accessions and interspecific hybrids with *Triticum aestivum*	Iron, Zinc	Rawat et al., 2009a
18.	*Ae. geniculata* (MMUU)	DAL 5 M^g^	Iron, Zinc	Wang S. et al., 2011
19.	*Ae. biuncialis* (U^b^U^b^M^b^M^b^)	DSLs 3M^b^(4B), Translocation line 3M^b^.4BS	Potassium, Zinc, Iron, Manganese	Farkas et al., 2014
20.	*Ae. kotschyi* (UUSS)	Not mentioned	Iron, Zinc	Chhuneja et al., 2006
21.	*Ae. kotschyi* (UUSS)	DALs	Iron, Zinc	Rawat et al., 2009a
22.	*Ae. kotschyi* (UUSS)	Accessions and interspecific hybrids with *Triticum aestivum*	Iron, Zinc	Rawat et al., 2009a
23.	*Ae. kotschyi* (UUSS)	Amphiploids	Iron, Zinc	Rawat et al., 2009b
24.	*Ae. kotschyi* (UUSS)	Amphiploids (AABBDDUkUkSkSk)	Macronutrients, Micronutrients	Tiwari et al., 2010
25.	*Ae. kotschyi* (UUSS)	DSLs 2S, 7U	Iron, Zinc	Tiwari et al., 2010
26.	*Ae. kotschyi* (UUSS)	DALs, DSL	Iron, Zinc	Rawat et al., 2011
27.	*Ae. kotschyi* (UUSS)	Hybrids	Iron, Zinc	Sheikh et al., 2018
28.	*Ae. kotschyi* (UUSS)	Hybrids with small alien introgression	Iron, Zinc	Verma et al., 2016a
29.	*Ae. kotschyi* (UUSS)	U/S introgression	Iron, Zinc	Verma et al., 2016b
30.	*Ae. kotschyi* (UUSS)	DSLs	Iron, Zinc	Sharma et al., 2018
31.	*Ae. kotschyi* (UUSS)	Hybrids	Iron, Zinc	Sharma et al., 2018
32.	*Ae. kotschyi* (UUSS)	Derivatives	Iron, Zinc	Sheikh et al., 2018
33.	*Ae. kotschyi*	Fine translocation line U/S	Iron, Zinc	Verma et al., 2016b
34.	*Ae. peregrina* (UUSS)	DALs	Iron, Zinc	Rawat et al., 2009a
35.	*Ae. peregrina* (UUSS)	Accessions and interspecific hybrids with *Triticum aestivum*	Iron, Zinc	Rawat et al., 2009a
36.	*Ae. peregrina* (UUSS)	DAL 4S^v^	Iron, Zinc	Wang S. et al., 2011
37.	*Ae. peregrina* (UUSS)	DALs 2S^v^, 2U^v^, 7U^v^	Iron, Zinc	Kumari et al., 2012
38.	*Ae. peregrina* (UUSS)	DSLs	Iron, Zinc	Sharma et al., 2018
39.	*Ae. peregrina* (UUSS)	Derivatives	Iron, Zinc	Sheikh et al., 2018
40.	*Ae. peregrina* (UUSS)	Hybrids	Iron, Zinc	Sharma et al., 2018

*DALs – Disomic addition lines, DSLs – Disomic substitution lines, SHW – synthetic hexaploid wheat*.

Disomic addition/substitution lines can be utilized to introgress useful variability of high grain Fe and Zn from *Aegilops* into wheat in form of short arm or fine chromosomal translocations through induced homoeologous pairing. Interspecific hybrids of *Ae. longissima* with *T. turgidum* (Tiwari et al., 2008) and *Ae. kotschyi* (Sheikh et al., 2018) produced after crossing addition /substitution lines with tetraploid and hexaploid wheat also showed elevated levels of grain iron and zinc content. *Ae. biuncialis* specific translocation line 3M^b^.4BS (Farkas et al., 2014) and many U/S chromosome specific fine translocations of *Ae. kotschyi* in wheat (Verma et al., 2016a,b) have been produced with least linkage drag effect. These lines also showed significant increase in grain iron and zinc content.

### Improvement in Phytochemicals Concentration

Studies on phytochemical contents of *Aegilops* species have been limited (Figure 3) with their work mainly being carried out in Europe (Figure 4). But given the rich genetic diversity of *Aegilops*, many phytochemicals such as phenolic acids, carotenoids, tocopherols, alkylresorcinols, benzoxazinoids, phytosterols and lignans can be explored in *Aegilops* species. Many phenolic diglycerides have been detected in *Ae. geniculata* (Cooper et al., 1978) (Table 6). p-hydroxybenzaldehyde, vanillin and mono-epoxylignanolide (MEL) have been detected in *Ae. geniculata* (Cooper et al., 1994). Alloplasmic lines derived from wheat and *Ae. squarossa* have been shown to increase the lutein content (Atienza et al., 2008). Synthetic hexaploid wheat (SHW) lines generated by crossing tetraploid durum wheat and *Ae. tauschii* also showed increased yellow pigment content and might be useful source for increasing carotenoids content in wheat (Li et al., 2015). DALs of *Ae. geniculata* and *Ae. biuncalis* showed increase in total protein content and polymeric proteins (Rakszegi et al., 2017) hence enhancing the nutritive value (Table 6).

**Table 6:** *Aegilops* species explored for phytochemicals and dietary fibers.

S.No.	Species	Source	Traits	Reference
1	*Ae. speltoides* (SS)	2140008	DIMBOA-glucoside	Elek et al., 2014
2	*Ae. crassa* (DDMM)	Recombinants of *Triticale* with *Ae. crassa*	Protein, dietary fiber, thousand kernel weight, volume weight	Boros et al., 2010
3	*Ae. geniculata* (MMUU)		Tricin and flavo-lignan	Cooper et al., 1977
4	*Ae. geniculata* (MMUU)		Scopoletin and p-coumaric acid	Cooper et al., 1978
5	*Ae. geniculata* (MMUU)	2U^g^, 4U^g^, 5U^g^, 7U^g^, 2M^g^, 5M^g^, 7M^g^ DALs	Protein content	Rakszegi et al., 2017
6	*Ae. geniculata* (MMUU)	1U^g^, 1M^g^ DALs	Polymeric glutenin proteins	Rakszegi et al., 2017
7	*Ae. geniculata* (MMUU)	5U^g^, 7U^g^ DALs	Arabinoxylan	Rakszegi et al., 2017
8	*Ae. biuncialis* (UUMM)	1U^b^ DAL	Arabinoxylan	Rakszegi et al., 2017
9	*Ae. geniculata* (MMUU)	5U^g^, 5M^g^,7M^g^ DALs	β-glucan	Rakszegi et al., 2017
10	*Ae. biuncialis* (UUMM)	3U^b^, 2M^b^, 3M^b^, and 7M^b^ DALs	Protein	Rakszegi et al., 2017
11	*Ae. biuncialis* (UUMM)	5U^b^, 5M^b^, 7M^b^ DALs	β -glucan	Rakszegi et al., 2017
12	*Ae. juvenalis* (DDMMUU)	Recombinants of *Triticale* with *Ae. juvenalis*	Protein, dietary fiber, thousand kernel weight, volume weight	Boros et al., 2010

*DALs – Disomic addition lines, DIMBOA – 2,4-dihidroxy-7-methoxy-1,4-benzoxazin-3-one*.

### Improvement in Dietary Fibers Concentration

Dietary fibe**r**s are important components of wheat which impact processing quality and have many health benefits. The major components of dietary **fibers** in wheat grain are cell wall polysaccharides, arabinoxylan (AX) and (1-3)(1-4)- β-D-glucan (β-glucan). Both of these occur in soluble and insoluble forms with different health benefits such as reduced risks of type II diabetes, coronary heart diseases and prevention of colon cancer. Soluble forms of dietary **fibers** also include FODMAPs (Fermentable oligosaccharides, disaccharides, monosaccharides and polyols) which are a group short chain carbohydrates. A diet rich in FODMAPs is often associated with diseases like Crohn disease and irritable bowel syndrome (IBS), which is a chronic gastrointestinal disease (Khan et al., 2015). Dietary **fiber** components have been reported to affect processing quality of wheat in terms of bread making and starch gluten separation. Arabinoxylan have effects on water absorption and development time of dough (Courtin and Delcour, 1998). β-glucan confers high viscosity, higher water absorption, lower loaf volume, height and stiffer dough (Symons and Brennan, 2004; Cleary et al., 2007; Skendi et al., 2009). From nutrition point of view higher levels of β-glucan are sought in food products as they lower serum cholesterol levels and regulate glucose levels in blood (McIntosh et al., 1991; Cavallero et al., 2002). Variability and composition of dietary **fiber**s have been extensively studied in wheat and related cereal grains. Wheat primary gene pool has been explored in the European HEALTHGRAIN cereal diversity screening project^1^ for dietary **fiber**s and other phytochemicals. However, such studies in wild species of wheat have been limited. There have been reports of recombinants of *Triticale* with *Ae. crassa* and *Ae. juvenalis* showing higher dietary **fiber** content along with increased values of total protein content, thousand kernel weight and volume weight (Boros et al., 2010) (Table 6). Both the species can be utilized for improving the nutrition value of wheat. Addition of 5U^g^, 7U^g^ chromosome pairs of *Ae. geniculata* and 1U^b^ of *Ae. biuncialis* into wheat have resulted in increased arabinoxylan content (Rakszegi et al., 2017). Similarly, addition of 5U^g^, 5M^g^, and 7M^g^ chromosome pairs from *Ae. geniculata* and 5U^b^, 5M^b^, and 7M^b^ chromosomes from *Ae. biuncialis* have been reported to result in elevated levels of β-glucan content in wheat (Rakszegi et al., 2017). Since there is a large genetic diversity available in *Aegilops* species, they need to be explored for dietary **fiber**s content and their potential use for enhancing nutritional value of wheat.

## Conclusion

Quality and nutrition are two very important aspects of wheat research. Over the past few years, a lot of emphasis has been given by breeders worldwide to improve the end product quality of wheat and to develop varieties that meet specific end product and nutritional requirements. New sources of genetic variations in wheat are always sought after because of the narrow genetic diversity. Wild species of wheat can serve as excellent source of new variations that can be incorporated into wheat. Close relatedness to wheat makes *Aegilops* the most favorable genetic resource for wheat improvement through alien gene introgression. The basic approach for alien gene transfer is to cross the wild relative with wheat to generate interspecific hybrids followed by embryo rescue and colchicine treatment to double chromosomes. The amphiploids generated are then backcrossed multiple times with wheat to generate addition/substitution lines (Friebe et al., 1995, 1996, 1999). A large number of wheat-*Aegilops* amphiploids and chromosome addition/substitutions lines are available (Schneider et al., 2008). But these addition/substitution lines and amphiploids have no practical application in agriculture as the *Aegilops* chromosome segment carrying the gene of interest must be transferred to the wheat chromosome as translocation. The *Ph1* locus, present at the long arm of chromosome 5B regulates chromosome pairing in wheat and ensures that only homologous chromosomes pair at metaphase. To generate translocations between wheat chromosome and alien chromosome, *Ph1* mutants or *Ph1* suppressors can be used to bypass the *Ph1* control mechanism of homologous pairing. Translocations can also be generated via radiation induced chromosome breaks followed by random recombination. The recombinants generated then need to be screened using chromosome pairing, C banding pattern and *in situ* hybridization. Thus, the whole process of alien gene transfer is laborious and time consuming. However, with technological advancements and development of new high throughput marker technologies it is now possible to identify desirable recombinants from a large population with great precision and efficiency (Niu et al., 2011; Tiwari et al., 2014).

A large number of countries throughout the world are participating in the exploration of *Aegilops*. HMW GS are most explored, while phytochemicals are least explored among different research groups across the world. Gliadins have been explored by highest number of countries while, LMW GS and phytochemicals are least explored around the world (Figure 3). Based on this review we are aware that more than 95 subunits of HMW GS, 51 novel alleles of LMW GS, 34 alleles for *Pin a*, 40 alleles for *Pin b* and 26 alleles for *GSP* in *Aegilops* have been reported across multiple accessions, synthetic lines, addition/substitution lines and translocation lines (Figure 3). These can serve as excellent genetic sources of variation for wheat quality improvement. Large numbers of publications have arisen for *Aegilops* exploration for improvement of nutrition and processing quality. Highest exploration has been carried out in China and Europe followed by Japan and India (Figure 4). Major work on LMW GS has been carried out in China, Fe/Zn in India, others having good distribution across countries (Figure 4). More than 14 species of *Aegilops* have been proven to be excellent sources for the improvement of grain micronutrient content, protein content, dietary fiber content and phytochemical content. Many *Aegilops* species have already been incorporated in various breeding programs across the world. Still there is further need to explore *Aegilops* species to identify new variations. Though a large number of accessions are available in gene banks, many accessions of *Aegilops* species still remain unexploited. The real bottleneck for introgressing useful genes into wheat from *Aegilops*, however, is the generation of fine translocation lines containing the smallest possible segment of alien chromosome with the gene of interest. Although a lot of scientific exploration has been carried out, practically we still are nowhere in terms of introgressing and utilizing genes related to quality and nutrition from *Aegilops* species. There is still a long way to go. It is anticipated that the availability of the newly annotated wheat genome sequence (International Wheat Genome Sequencing Consortium, Appels et al., 2018) along with new genomic tools and genetic resources will aid the further exploration and exploitation of *Aegilops* species and the transfer of useful traits into wheat.

## Author Contributions

AK and MG built the layout of article. AK, MG, and PK collected the literature. AK wrote the article. MG, VC, SS, and PK helped in manuscript editing. PK did the reference management. All authors prepared images and tables.

## Conflict of Interest Statement

The authors declare that the research was conducted in the absence of any commercial or financial relationships that could be construed as a potential conflict of interest.

## Footnotes

1 https://healthgrain.org
